# The effects of cocoa products in individuals with metabolic syndrome and related diseases: a systematic review and meta-analysis

**DOI:** 10.1007/s40200-026-01914-7

**Published:** 2026-03-17

**Authors:** Amanda Gomes Chagas, Bruno Giusti Camilotti, Gabriela Nascimento Gonçalves, Leandro Roberto de Macedo, Maísa Silva

**Affiliations:** 1 Universidade Federal de Juiz de Fora, Campus Governador Valadares, Governador Valadares, Minas Gerais Brazil; 2 Department of Economy, Universidade Federal de Juiz de Fora, Governador Valadares, Brazil; 3 Department of Basic Life Sciences, Universidade Federal de Juiz de Fora, Governador Valadares Campus, Av. Moacir Paleta, 1167 - São Pedro, Gov. Valadares, MG 35020-360 Brazil

**Keywords:** Randomized clinical trials, Cocoa, Metabolic syndrome, Meta-analysis

## Abstract

Cocoa supplementation has been shown to improve parameters related to metabolic syndrome, although results have been contradictory. We conducted a systematic review and meta-analysis to examine the effect of cocoa products on lipid and glycemic profiles, blood pressure levels, and anthropometric measurements in individuals with metabolic syndrome and related diseases. A search of PubMed, Web of Science, Embase, and Scopus was performed to identify randomized clinical trials (RCTs). To compare the effects of the cocoa product with placebo, mean differences with 95% confidence intervals (CIs) were pooled using a random-effects model. Subgroup analyses and meta-regression were performed to identify the source of heterogeneity, and study quality assessment was conducted using the GRADE approach. A total of 13 RCTs, including 16 arms, that investigated the effects of cocoa on parameters related to metabolic syndrome were included. The meta-analysis showed a significant effect of cocoa supplementation on triacylglycerol levels (-0.21 mmol/L, 95% CI: -0.40, -0.02; *p* = 0.0333), and subgroup analysis suggested that cholesterol levels were reduced with cocoa supplementation in patients with dyslipidemia and diabetes. Furthermore, the meta-regression analysis suggested a decrease in glycemic and lipid profiles, as well as anthropometric parameters, in studies with longer durations of cocoa supplementation. Our study suggests that cocoa intake has limited evidence of benefit, mainly for triglycerides, furthermore, effects and that the duration, type, and clinical status of patients were important determinants for favorable effects on biomarkers related to metabolic syndrome.

**Supplementary Information:**

The online version contains supplementary material available at 10.1007/s40200-026-01914-7.

## Introduction

Metabolic syndrome (MetS) is characterized by the coexistence of cardiovascular risk factors and metabolic disorders, such as hypertension, insulin resistance, dyslipidemias – elevated triglyceride levels and reduced HDL levels – and increased waist circumference [1, 2]. The presence of MetS is a risk factor for other conditions, particularly increasing the risk of developing type 2 diabetes (DM2) by five times and the risk of cardiovascular diseases by three times [3]. Data from the International Diabetes Federation indicate that the prevalence of MetS in the Americas (33.4%) is the second highest in the world, surpassed only by the Eastern Mediterranean region (34.6%) [4]. It may be the result from a combination of genetic factors, dietary profile, and physical inactivity [5].

Strategies such as adhering to a healthy dietary pattern can improve metabolic profile and thus reduce the risk of developing metabolic syndrome and associated diseases [6]. Furthermore, studies have shown that functional foods, probiotics, dietary supplements, and trace elements can help prevent cardiovascular disease by improving the lipid profile, blood pressure, and oxidative stress [7–11]. Cocoa is a food rich in flavonoids and the consumption of these compounds can reduce the risk of developing DM2 and Non-Alcoholic Fatty Liver Disease (NAFLD) [12, 13]. Studies show that individuals who consume cocoa and products derived have smaller waist circumference, fasting blood glucose [12] and reduced the risk of incidence of hypertension [14]. However, the consumption of dark chocolate in individuals with mild hypertension promote a reduction in diastolic blood pressure, but no significant effects on vascular function, markers of glucose/lipid metabolism, renin–angiotensin–aldosterone system, and oxidative stress [15]. Results found in meta-analyses on the effect of cocoa on metabolic parameters also present discrepancies, as in the study by Arisi et al. in 2024 [16], cocoa showed positive effects on total cholesterol, LDL, fasting glucose, SBP and DBP, but did not produce a reduction effect on triglycerides, body weight, BMI, waist circumference, HDL cholesterol and glycated hemoglobin (HbA1c) in adults, with and without comorbidities. On the other hand, the meta-analysis produced by Chen et al. in 2022 [17], which found that consumption of cocoa products significantly reduced LDL cholesterol, triglycerides and glycemia in patients with type 2 diabetes.

Despite some publications on the effects of cocoa products on MetS-related parameters, we know of no meta-analysis in individuals with MetS and related diseases. However, several recent meta-analyses of bioactive plant-derived foods in closely related metabolic conditions (e.g., non-alcoholic fatty liver disease, hypertension, insulin resistance) provide relevant mechanistic and clinical context [18–20]. For example, a meta-analysis of Chlorella vulgaris in NAFLD [21] reported improvements in glycemia, lipids, and liver enzymes, but with very low certainty due to bias and heterogeneity. Thus, this study aims to conduct a systematic review and meta-analysis of randomized controlled trials to evaluate the hypothesis that cocoa and its products have an ameliorating effect on parameters such as blood pressure, lipid and glycemic profile, and anthropometric parameters in individuals with MetS and related disorders.

## Materials and methods

The systematic review was conducted and reported according to the Preferred Reporting Items for Systematic reviews and Meta-Analyses (PRISMA) guidelines [22], and PRISMA 2020 check list [23]. The review has been registered at PROSPERO international prospective register of systematic reviews CRD42024586659.

### Search strategy and selection criteria

We used the PICOS model to determine the inclusion criteria standing for population (aged > 18 years old with metabolic syndrome or related disorders), intervention (cocoa supplementation), comparison (control group), outcome (MetS risk factors) and study (randomized controlled trials). Medline, Scopus, Web of Science and Embase databases were searched using the following search terms in titles and abstracts (also in combination with MeSH terms): “cocoa” OR “cacao” OR “chocolate” AND “blood pressure OR hypertension” OR “lipid” OR “cholesterol” OR “triglyceride” OR “blood glucose” AND “randomized controlled trial”. The search was limited to studies published in the last ten years. A manual review of the reference lists and citations of each identified study was also conducted. Literature searches were conducted until September, 2024. When applicable, attempts were also made to contact investigators for clarification or additional unpublished data. No language restrictions were imposed.

The search was performed independently by three authors (AGC, BGC and GNG). In case of disagreement, a fourth investigator was consulted (MS). The agreement between reviewers regarding the classification of the studies was assessed using the Cohen's kappa coefficient [24].

### Inclusion/exclusion criteria

All clinical trials were then entered for final meta-analysis if they had the following criteria: (I) human trials with either crossover design or parallel; (II) the subjects in the trial were exposed to the intervention for a minimum of 1 weeks; (III) reported the impact of cocoa supplementation on blood pressure, lipid profiles, blood glucose, weight, body mass index (BMI), waist circumference (WC), glycated hemoglobin (HbA1c), insulin, and homeostasis model assessment insulin resistance index (HOMA-IR) at baseline and follow-up; (IV) performed in adult subjects; (V) patients with metabolic syndrome or related disorders. In this meta-analysis, letters, short communications, reviews, animal studies and in vitro were excluded from the analysis. Duplicate studies, trials without sufficient data and the intervention used a mixture of cocoa and other substances were also excluded. Studies with cocoa supplementation and physical exercise or lifestyle intervention were not included in this review. Trials evaluating multiple treatment arms (low- or high-dose cocoa) were included in the meta-analysis as a separate trial.

### Data extraction and quality assessment

Eligible studies were reviewed and the following data were abstracted: study characteristics (authors and publication year), study design, population information, the dose and type of cocoa supplementation, the duration of the study, health condition, and MetS risk factors (main outcomes). A pre-structured Excel 2019 database divided into columns was used to compile the extracted data. The selection of studies and data extraction were performed by three authors (AGC, BGC, and GNG), and supervised by another researcher (MS). Any discrepancies between reviewers were resolved by consensus.

The tool used to assess the risk of bias was revised Cochrane risk-of bias tool for randomized trials (RoB 2) [25]. Four reviewers (AGC, BGC, GNG, and MS) independently assessed the risk of bias of the included randomized clinical trials. Disagreements between investigators were resolved by consultation with the senior investigator (MS). Quality was assessed according to the following criteria: randomization process, deviations from intended interventions, missing outcomes data, measurement of outcomes, and selection of the reported results. Each domain was graded (low, high, or some concerns) based on the available information in the study. An overall risk of bias was assigned to each publication as recommended in the guidelines. In the case of a crossover study, the questions in each domain were adapted according to the Cochrane guidelines [25]. All disagreements were resolved by discussion. The criteria used for quality assessment were described in the Cochrane Collaboration tool [25].

We assessed the quality of evidence for each category using the Grading of Recommendations Assessment, Development and Evaluation (GRADE) approach [26]. We rated the quality of evidence of the outcomes across trials using GRADE-provided criteria, including study risk-of-bias, inconsistency, indirectness, imprecision, and publication bias. In meta-analyses using only RCTs, the initial recommendation is: to classify the study design as "high". GRADE categorized the quality of evidence into four levels: High, Moderate, Low and Very Low quality. For the risk of bias, a score was generated based on the findings of the Cochrane risk-of bias tool. The "inconsistency" item was evaluated by the similarity of the effect estimates with a 95% CI overlapping, as well as by the degree of heterogeneity (I^2^). For the "indirectness" the similarities between the participants, interventions, and outcomes evaluated were considered. Using forest plots with wide 95% CIs for each study and outcome, "imprecision" was visually assessed. The "risk of publication bias" was assessed by the symmetry of the funnel plot and Egger test.

### Statistical analysis

For each factor, we extracted the mean at baseline and post-intervention, from both the intervention and control groups. Standard deviations (SDs) of the mean differences were calculated using the following formula:

$$SD=$$$$\sqrt{\begin{array}{c}{\left(S{D}_{pre-treatment}\right)}^{2}+{\left(S{D}_{post-treatment}\right)}^{2}\\-2R\times S{D}_{pre-treatment}\times S{D}_{post-treatment}\end{array}}$$, assuming a correlation coefficient (R) = 0.5 [27–29]. We performed sensitivity analyses using plausible alternative values for the correlation coefficient (r = 0.25 and r = 0.75). For data extraction from eligible studies with results presented in graphs, we used GetDate Graph Digitizer 2.26 [30] to extract them. When the unit of variance was reported as standard error or confidence interval, conversion into SD was conducted in accordance with the Cochrane guidelines [28]. The meta-analysis was performed using R software (R Foundation for Statistical Computing, Vienna, Austria, URL http://www.R-project.org, 2020).

Statistic heterogeneity of treatment effects between studies was formally tested with Cochrane’s test [31]. The I^2^ statistic was also examined, and we considered an I^2^ value > 50% and > 75% to indicate substantial and considerable heterogeneity, respectively, between the trials. Based on the heterogeneity between included studies, a random effect or a fixed model was applied in the meta-analysis. The random-effects model was selected a priori based on conceptual and methodological considerations, including the expected clinical and methodological variability among the included studies. The pooled effect size estimated using the DerSimonian-Laird Based random-effects model [32]. To investigate the potential sources of between-study heterogeneity, we carried out a subgroup analysis, recognizing the risk of type I error inflation, based on age of participants of studies as proposed by Akhlaghi [33]. Dosage, type of cocoa supplementation, and the duration of treatment as proposed by Vlachojannis et al. [34]. The design of studies, gender of participants of studies, and presence or absence of dyslipidemia and diabetes as proposed by Tanghe et al. [35].

Effect sizes were presented as mean differences with 95% confidence intervals, and *p*-values < 0.05 were considered as statistically significant [36]. Publication bias was assessed by the Egger’s test and represented graphically by funnel plots [37–39]. Sensitivity analyses were also performed by removing 1 study at a time, to assess any impact of study quality on the effect estimates [40]. Meta-regression analysis was carried out to investigate the association between the duration of the intervention and the age mean with pooled effect size [41].

## Results

### Search results

**Fig. 1:**
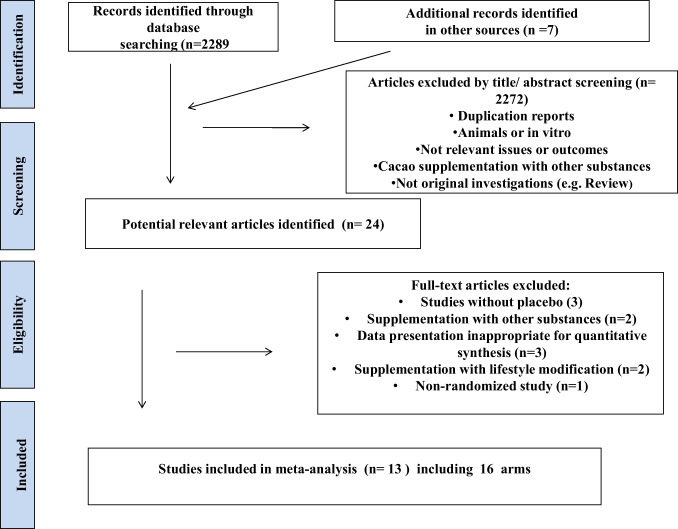
Literature search and review flow chart for selection of studies

An overall of 2289 studies were retrieved through initial online database search and 7 additional records identified in other sources. After removing that did not meet the inclusion criteria, 24 references remained. These potentially relevant articles were examined for full text evaluation. The other 11 articles were excluded for the following reasons: data presentation inappropriate for quantitative synthesis in three trials, two articles with cocoa supplementation and other substances were discarded, three studies without placebo, two articles were supplementation and lifestyle modification and one non-randomized study. Thus, 13 studies were included in the meta-analysis, including 16 arms (Fig. 1). In trials with multiple doses of cocoa, the control group was divided to avoid double counting of participants. Cohen's Kappa analyses showed values ​​ranging from 0.64 to 0.91, all statistically significant (*p* < 0.001), demonstrating a high degree of consistency among reviewers in classifying the studies.

The main characteristics of trials included in this meta-analysis are summarized in Table 1. The selected studies enrolled subjects with different health conditions, five studies addressed overweight patients [42–44], being one of them also with borderline criteria of metabolic syndrome [45] and one with pre-menopausal females with overweight/obesity [46]. Three studies included adults with diabetes, of those, one with type 2 diabetes [47], one with type 2 diabetes with hypertension [48] and one with diabetes and hypertension [49]. Besides those, prediabetic patients were selected by one study [50]. Nonalcoholic fatty liver disease was addressed by one study [51], as well as stage 1 hypertension [52], metabolic syndrome [53] and smokers with cardiovascular comorbidities [54].

**Table 1:** Characteristics of included studies

First author (publication year)	Design	Number of participants/Gender	Mean age (years)	Type and amount of cocoa intake	Duration	Notes about participants	Main outcomes
Gomes et al*.* 2023	Parallel, no blind	Placebo = 17 Cocoa = 15	No reported	40 g of chocolate containing 70% cocoa/day	4 weeks	smokers with cardiovascular comorbidities	Weight, BMI, Insulin
Simpson et al*.* 2023	double-blind, parallel	Placebo = 16 Cocoa = 16	Placebo = 34.8 ± 9.13 Cocoa = 31.9 ± 11.20	high-flavanol cocoa (609 mg cocoa flavanols, 95 mg (-)-epicatechin)	4 weeks	Pre-menopausal females with overweight/obesity	Weight, Insulin
Leon- Flores et al*.* 2020	Blind, parallel	Placebo = 11 Cocoa = 13	Placebo = 42 ± 2.2 Cocoa = 48.3 ± 1.9	cookies with flavonoids extracted from the cocoa containing 12.5 mg of EPI equivalents, twice a day	8 weeks	overweight	Weight, BMI, WC, glucose, TC, TG, LDL, HDL,
Shiina et al*.* 2019 (1)	double-blind, crossover	11	59.3 ± 7.1	cocoa procyanidin supplement (83.3 ± 2.7 mg/day)	4 weeks	Males prediabetic	LDL, HOMA, SBP, DBP
Shiina et al*.* 2019 (2)	double-blind, crossover	11	59.3 ± 7.1	cocoa procyanidin supplement (83.3 ± 2.7 mg/day)	4 weeks	Females prediabetic	LDL, HOMA, SBP, DBP
Dicks et al*.* 2018	double-blind, parallel	Placebo = 18 Cocoa = 17	Placebo = 62.8 ± 1.6 Cocoa = 65.6 ± 2.6	2.5 g/day of a flavanol-rich cocoa	12 weeks	Type 2 Diabetes and Hypertension	glucose, HbA1c, insulin, HOMA, weight, WC, SBP, DBP, TC, TG, LDL, HDL
Leyva-Soto et al. 2018	double-blind, parallel	Placebo = 42 Cocoa = 42	Placebo = 23.6 ± 3.5 Cocoa = 23.8 ± ± 3.4	2 g of dark chocolate containing 70% cocoa	6 months	Metabolic syndrome	glucose, HbA1c, HOMA, BMI, WC, SBP, DBP, TC, TG, LDL, HDL
Njike et al*.* 2016 (3)	double-blind, parallel	Placebo = 26 Cocoa = 25	Placebo = 54.2 ± 10.1 Cocoa = 54.2 ± 10.1	10 g cocoa powder every day	8 weeks	stage 1 hypertension	glucose, insulin, HOMA, weight, BMI, WC, SBP, DBP
Njike et al*.* 2016 (4)	double-blind, parallel	Placebo = 26 Cocoa = 24	Placebo = 53.0 ± 10.6 Cocoa = 53.0 ± 10.6	5 g cocoa powder every day	8 weeks	stage 1 hypertension	glucose, insulin, HOMA, weight, BMI, WC, SBP, DBP, TC, TG, LDL, HDL
McFarlin et al*.* 2015 (5)	double-blind, crossover	Overweigh*t* = 7	22 ± 3	cocoa-containing product (12.7 g natural cocoa)	4 weeks	overweight	TC, TG, HDL, glucose
McFarlin et al*.* 2015 (6)	double-blind, crossover	Obese = 7	21 ± 3	cocoa-containing product (12.7 g natural cocoa)	4 weeks	obese	TC, TG, HDL, glucose
Munguía et al*.* 2015	double-blind, parallel	Placebo = 5 (2 males) Cocoa = 10 (2 males)	No reported	Cocoa flavonoids (80 mg)	4 weeks	overweight subjects with borderline criteria of metabolic syndrome	weight, BMI, WC, SBP, DBP, glucose, TC, TG, LDL, HDL
Alavinejad et al. 2015	double-blind, parallel	Placebo = 21 (18 males) Cocoa = 21 (15 males)	Placebo = 37.95 ± 10.34 Cocoa = 38.18 ± 11.04	30 gr dark chocolate (83%)	12 weeks	Nonalcoholic fatty liver disease	BMI, WC, glucose, TC, TG, LDL, HDL
Rostami et al*.* 2015	double-blind, parallel	Placebo = 28 (12 males) Cocoa = 32 (12 males)	Placebo = 57.17 ± 7.86 Cocoa = 58.71 ± 9.07	25 g dark chocolate	8 weeks	diabetes and hypertension	glucose, insulin, HbAc1, SBP, DBP, TC, TG, LDL, HDL
Parsaeyan et al*.* 2014	Parallel	Placebo = 50 Cocoa = 50	54 ± 5	20 g cocoa poder	6 weeks	type 2 diabetes	TC, TG, LDL, HDL
West et al*.* 2014	double-blind, crossover	30	51.7 ± 1.2	37 g/d of dark chocolate	4 weeks	overweight	insulin, HOMA, TC, TG, LDL, HDL,weight, BMI, WC

Abbreviations: *TG* triacylglycerol, *TC* total-cholesterol, *LDL* Low-density lipoprotein cholesterol, *HDL* High-density lipoprotein cholesterol, *HOMA-IR* homeostasis model assessment of insulin resistance, *BMI* Body mass index, *WC* Waist circumstance, *SBP* Systolic blood pressure, *DBP* Diastolic blood pressure

The shortest clinical trial was 4 weeks, while the longest trial was 12 weeks, with sample sizes ranging from 15 to 100 participants. The mean age of participants ranged from 21 to 65 years. Two studies recruited only women [44, 46] and seven studies did not report the proportion of men and women who were recruited and analyzed in the control and placebo groups [43, 50, 52–54]. Regarding the intervention method, one study used chocolate [54], four used dark chocolate [42, 49, 51, 53], two used cocoa with a high flavonol content [42, 44], one used cocoa flavonoids [41], two used cocoa powder [47, 52], one used cocoa products [44], one used cookies with flavonoids extracted from cocoa [43] and only one used a cocoa procyanidin supplement [50]. Cocoa dosages ranged from 2,5 to 28 g/day of cocoa.

Six RCTs, with eight arms, reported on SBP and DBP [44, 48–50, 52, 53], and twelve RCTs reported on blood lipid profiles, including nine RCTs, with ten arms, on TG and TC [42–45, 47–49, 51–53], nine RCTs, with ten arms, on LDL [42, 43, 45, 47–53], and nine RCTs, with ten arms on HDL [42–45, 47–49, 51–53]. Thirteen studies provided data on glycemic status markers, including seven RCTs, with eight arms reporting on FPG [42, 45, 48, 49, 52, 53], six RCTs, with seven arms on fasting insulin [42, 46, 48, 49, 52, 54], five RCT, with seven arms on HOMA-IR [42, 48, 50, 52, 53], and three on HbA1c [48, 49, 53]. Furthermore, nine studies, with ten arms, reported anthropometric measurements, such as BMI (seven RCTs, with eight arms) [42, 43, 45, 48, 51–55], WC (seven RCTs, with eight arms) [42, 43, 45, 48, 51–53], and weight (seven RCTs, with eight arms) [42, 43, 45, 46, 48, 52, 54].

### Risk of bias assessment

**Fig. 2:**
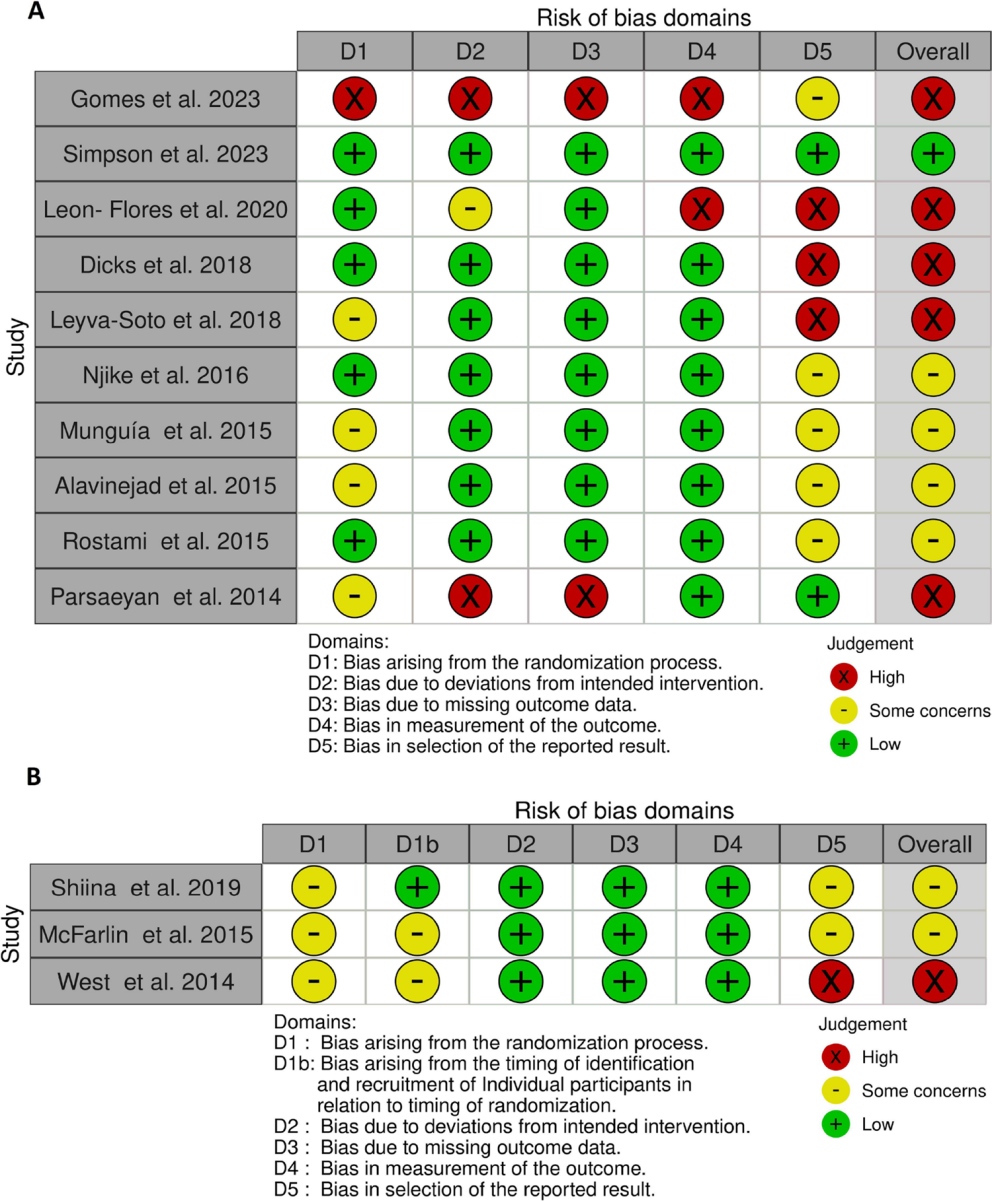
The summary of review authors' judgments about each risk of bias item for included studies

The Cochrane bias evaluation was performed to evaluate study and reporting quality are shown in Fig. 2. Five studies provided comprehensive explanations from the randomization process [43, 44, 48, 49, 52]. Two trials were considered high risk of bias due to deviations from intended intervention and due to missing outcome data [47, 54]. Two studies were showed high risk of bias due measurement of the outcomes [43, 54]. In four trials high risk of bias was found in selection of the reported result [42, 43, 48, 53].

### Quantitative analysis

#### Effect of cocoa supplementation on blood lipid profiles

**Fig. 3:**
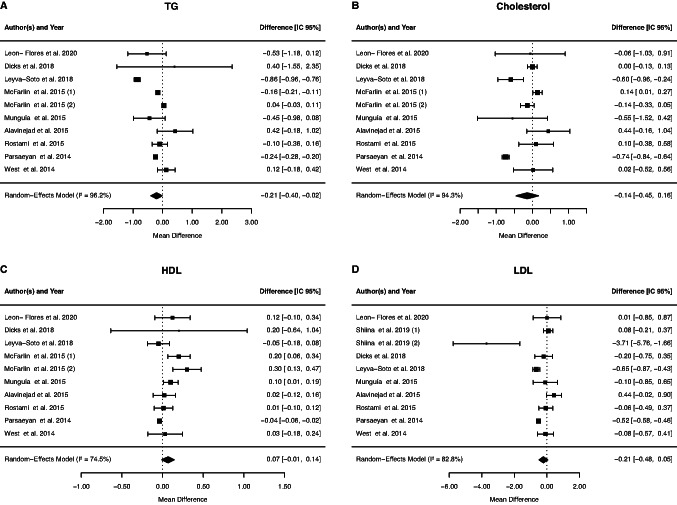
Forest plot of the effect of cocoa supplementation on blood lipid profiles

The effects of cocoa supplementation in ten studies were analysed, and meta-analysis did not verify a significant change in TC levels (−0.14 mmol/L, 95% CI: −0.45, 0.16; *p* = 0.3606), with between-study heterogeneity (*p* = < 0.0001, I2 = 94.31%, τ^2^ = 0.44, Cochran's Q test *p* = < 0.0001). The meta-analysis of ten studies did not verify a significant effect of cocoa supplementation on HDL levels (0.07 mmol/L, 95% CI: −0.01, 0.14; *p* = 0.0876), with between-study heterogeneity (*p* = < 0.0001, I2 = 74.46%, τ^2^ = 0.09, Cochran's Q test *p* = < 0.0001). Meta-analysis of ten studies revealed no significant effect on serum LDL levels when cocoa was administered (−0.21 mmol/L, 95% CI: −0.48, 0.05; *p* = 0.1170), with significant between-study heterogeneity (*p* = < 0.0001, I2 = 82.83%, τ^2^ = 0.34, Cochran's Q test *p* = < 0.0001). Ten RCTs were used to investigate TG, and a significant effect of cocoa supplementation was found by meta-analysis (−0.21 mmol/L, 95% CI: −0.40, −0.02; *p* = 0.0333), with between-study heterogeneity (*p* = < 0.0001, I2 = 96.23%, τ^2^ = 0.25, Cochran's Q test *p* = < 0.0001) (Fig. 3).

Subgroup analysis suggested that cocoa supplementation decreases cholesterol levels in patients with dyslipidemia and diabetes and in individuals who were supplemented with cocoa compared to studies with chocolate (Table 2). However, the duration and design of the study, the dosage used, the mean age and gender of the patients did not influence the levels of cholesterol (data not shown). We also performed subgroup analysis for HDL cholesterol levels. The subgroup analysis suggested increased HDL levels in studies with a crossover design, and in patients who did not have dyslipidemia or diabetes. Surprisingly, HDL levels decreased in parallel-design studies, individuals over 50 years of age, and diabetics (Table 2). However, the duration of the study, the dosage and type of cocoa used and gender of the patients did not influence the levels of HDL cholesterol (data not shown).

**Table 2:** The results of subgroup analyses

Subgrouped	No. of trials	Effect size (95% CI)	*P* Value	I^2^ (%)		*P* heterogeneity	*P* for between subgroup heterogeneity
Cholesterol							
Type							< 0.001
Cocoa	6	−0.20(−0.39, −0.01)	0.0311	81,9%		< 0.001	
Chocolate	4	−0.05 (−0.47, 0.37)	0.8075	67.3%		0.0098	
Dyslipidemia							< 0.001
Yes	2	−5.19 (−0.93, −0.26)	0.0005	0%		0.9337	
No	8	0.05 (−0.04, 0.16)	0.3267	84.4%		< 0.001	
Diabetes							< 0.001
Yes	5	−0.35 (−0.64, −0.06)	0.0164	87.2%		< 0.001	
No	5	−0.12 (- 0.27, 0.02)	0.2746		0%	0.1152	
HDL							
Study design							0.0563
Crossover	3	0.18 (0.03, 0.34)	0.0190		57.7%	0.1569	
Parallel	7	−0.03 (−0.05, −0.01)	0.0066		0%	0.0756	
Mean age							0.0472
< 50 years	5	0.11 (−0.01, 0.23)	0.0713		65.7%	0.0123	
> 50 years	4	−0.04 (−0.06, −0.02)	0.0006		0%	0.6968	
Dyslipidemia							0.0065
Yes	4	0.01 (−0.06, 0.08)	0.8396		56.9%	0.0173	
No	6	0.09 (0.03, 0.16)	0.0038		0%	0.0493	
Diabetes							0.0753
Yes	3	−0.04 (−0.06, −0.02)	0.0005		0%	0.5881	
No	7	0.09 (0.01, 0.18)	0.0240		55.3%	0.040	
LDL Study design							0.1013
Crossover	3	−1.05 (3.47, 1.36)	0.3910		98.1%	0.0015	
Parallel	7	−0.26(−0.50, −0.03)	0.0291		69.7%	0.0003	
Dyslipidemia							0.0073
Yes	4	−0.51(−0.70, −0.33)	< 0.001		44.6%	0.2600	
No	6	−0.41 (−1.63, 0.81)	0.5107		97.4%	0.0046	
TG							
Study design							< 0.001
Crossover	3	−0.03 (−0.18 0.12)	0.6859		87.92%	< 0.0001	
Parallel	7	−0.35 (−0.56, −0.14)	0.0010		85.21%	< 0.0001	
Mean age							< 0.001
< 50 years	5	−0.23 (−0.66, 0.19)	0.2785		98.80%	< 0.0001	
> 50 years	4	−0.22 (−0.27, −0.18)	< 0.0001		0%	0.0825	
Dyslipidemia							< 0.001
Yes	2	−0.73 (−1.10, −0.36)	< 0.0001		55.34%	0.1345	
No	8	−0.15(−0.18, −0.13)	< 0.001		0%	< 0.001	
Diabetes							< 0.001
Yes	5	−0.32(−0.35, −0.28)	< 0.001		95.84%	< 0.001	
No	5	−0.01 (−0.30, 0.27)	0.9241		97.4%	0.0046	
Glucose							
Type							< 0.001
Cocoa	4	0.16 (−0.18, 0.51)	0.3559		54.4%	0.0150	
Chocolate	3	−0.64 (−1.32, −0.05)	0.0350		80.37%	0.0002	
BMI							
Type							0.0399
Cocoa	4	−0.06 (−0.30, 0.17)	0.6045		0%	0.6594	
Chocolate	4	−1.18 (−2.32, −0.05)	0.0408		28.6%	0.0089	
DBP							0.2858
Cocoa	6	−0.30(−3.29, 2.68)	0.8412		61.18%	0.2570	
Chocolate	2	−4.71 (−7.73, −1.68)	0.0023		0%	0.3555	
WC							
Duration							0.005
< 8 weeks	3	0,323 (−2.69 3.32)	0.8343		0%	0.5205	
> 8 weeks	6	−3.00 (−4.09, −1.90)	<.0001		0%	0.0002	
Mean age							0.3370
< 50 years	3	−5.77 (−7.43, −4.10)	< 0.0001		0%	0.8356	
> 50 years	4	−0.60 (−1.94, 0.73)	0.3757		0%	0.0825	
Dyslipidemia							0.7246
Yes	3	−5.71 (−7.57, −4.24)	< 0.0001		0%	0.2740	
No	5	−0.54(−1.86, 0.76)	0.4138		0%	0.9013	

Subgroup analyses of LDL cholesterol levels suggested that these levels were reduced in studies with parallel and crossover designs and in diabetic patients (Table 2). On the other hand, the duration of the study, the dosage and type of cocoa used, gender and presence of dyslipidemia did not influence the levels of LDL cholesterol (data not shown). TG levels were decreased in a parallel-design study in individuals under 50 years of age, with diabetes and with and without dyslipidemia (Table 2). The duration of the study, the dosage and type of cocoa used and gender did not influence the levels of TG (data not show).

#### Effect of cocoa supplementation on markers of glycemic status

**Fig. 4:**
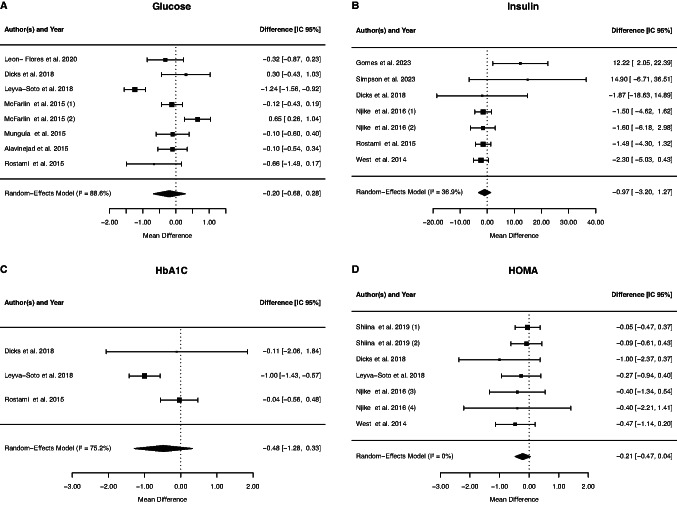
Forest plot of the effect of cocoa supplementation on markers of glycemic status

Eight trials were used to evaluate the effect of cocoa supplementation on FPG, and no significant changes were found (−0.20 mmol/L; 95% CI: −0.68, 0.28; *p* = 0.4106), with between-study heterogeneity (*p* = < 0.0001, I2 = 88,58%, τ^2^ = 0.63, Cochran's Q test *p* = < 0.0001). Seven RCTs were used to measure insulin, and no significant change was observed (−0.97 mU/L; 95% CI: −3.20, 1.27; *p* = 0.3969). The between-study heterogeneity was not significant (*p* = 0.1471, I2 = 36.87%, τ^2^ = 1.69, Cochran's Q test *p* = 0.15). In addition, seven RCTs used in this review with no heterogeneity among them (*p* = 0.8306, I2 = 0%, τ^2^ = 0, Cochran's Q test *p* < 0.0.83) demonstrated that HOMA-IR was not significantly influenced by cocoa supplementation (−0.21; 95% CI: − 0.47, 0.04; *p* = 0.0962). The meta-analysis of HbA1c parameters was performed with three studies; however, there was no significant effect of cocoa supplementation (−0,48%; 95% CI: − 1,28, 0.33; *p* = 0.2434), with between-study heterogeneity (*p* = 0.0178, I2 = 75.19%, τ^2^ = 0.57, Cochran's Q test *p* = 001) (Fig. 4).

Subgroup analysis suggested that chocolate supplementation decreased glucose levels compared to studies that supplemented with cocoa (Table 2). On the other hand, the duration and design of the study, the dosage, gender or mean age and presence of dyslipidemia or diabetes did not influence the levels of glucose (data not shown).

#### Effect of cocoa supplementation on blood pressure

**Fig. 5:**
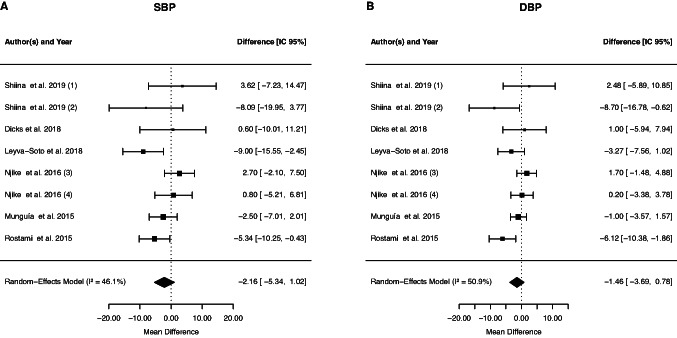
Forest plot of the effect of cocoa supplementation on blood pressure

The overall results of the meta-analysis of eight studies investigating the effect of cocoa supplementation on SBP did not show a significant change (− 2.16 mmHg, 95% CI: −5.34, 1.02; *p* = 0.1839) and no between-study heterogeneity (*p* = 0.077, I2 = 46.15%, τ^2^ = 2.99, Cochran's Q test *p* = 0.07). In addition, DBP was also not significantly changed (− 1.46 mmHg, 95% CI: −3.70, 0.78; *p* = 0.2015). The between-study heterogeneity was significant (*p* = 0.04, I2 = 50.90%, τ^2^ = 2.19, Cochran's Q test *p* = 0.04) (Fig. 5). Subgroup analysis suggested that chocolate supplementation decreased DBP compared to studies that supplemented with cocoa (Table 2). However, the duration and design of the study, the dosage, gender or mean age and presence of dyslipidemia or diabetes did not influence the DBP (data not shown).

#### Effect of cocoa supplementation on anthropometric measures

**Fig. 6:**
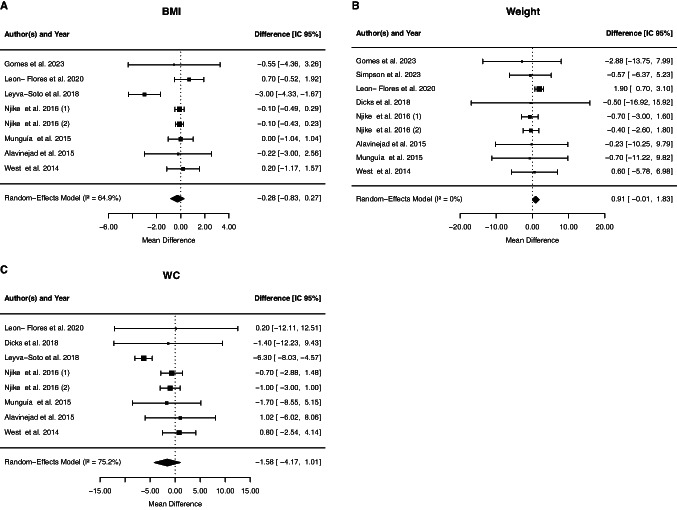
Forest plot of the effect of cocoa supplementation on anthropometric measures

Eight studies assessed the effect of cocoa supplementation on body weight, and no effects were observed (0.91 kg, 95% CI: −0.01, 1.83; p = 0.0532). The between-study heterogeneity was not significant (*p* = 0.5621, I2 = 0%, τ^2^ = 0, Cochran's Q test *p* = 0.56). The meta-analysis of eight studies did not show a significant effect of cocoa administration on WC (−1.58 cm, 95% CI: −4.14, 1.01; *p* = 0.2312), with between-study heterogeneity (*p* = 0.0002, I2 = 75.23%, τ^2^ = 2.81, Cochran's Q test *p* = 0.0002). Eight studies investigated the effect of cocoa supplementation on BMI. The pooled effect size did not show any significant effect on BMI (−0.28 kg m^−2^, 95% CI: − 0.82, 0.27; *p* = 0.3195), with between-study heterogeneity (*p* = 0.0056, I2 = 64.95%, τ^2^ = 0.54, Cochran's Q test *p* = 0.006) (Fig. 6).

Subgroup analysis suggested that chocolate supplementation decreased BMI compared to studies that supplemented with cocoa (Table 2). However, the duration and design of the study, the dosage, gender or mean age and presence of dyslipidemia or diabetes did not influence the BMI (data not shown). Waist circumference was reduced in studies lasting more than 8 weeks and in individuals under 50 years of age and with dyslipidemia (Table 2). The design of the study, the dosage and type, gender and presence of diabetes did not influence the WC (data not shown).

### Meta-regression

The meta-regression analysis was conducted to evaluate whether the changes in outcomes in response to cocoa supplementation could be associated with duration of intervention and age mean. The effect of cocoa supplementation on outcomes was independent of duration of intervention and age mean in SBP (*p* = 0.095 and *p* = 0.1572, respectively), DBP (*p* = 0.7524 and *p* = 0.7548, respectively), insulin (*p* = 0.3869 and *p* = 0.6552, respectively), HOMA (*p* = 0.6205 and *p* = 0.7394, respectively), total cholesterol (*p* = 0.6465 and p = 0.9795, respectively), HDL cholesterol (*p* = 0.1820 and *p* = 0.0509, respectively), LDL cholesterol (*p* = 0.6291 and *p* = 0.8763, respectively), and weight (*p* = 0.5791 and *p* = 0.0845, respectively).

**Fig. 7:**
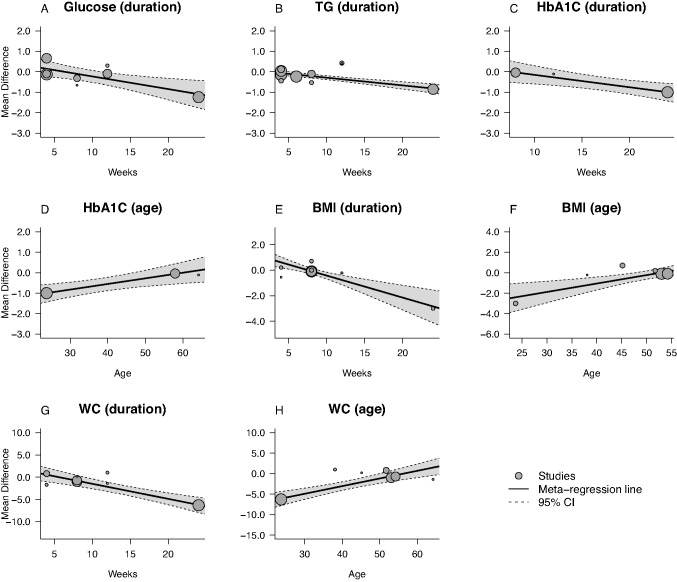
Meta-regression plots of the association of mean changes in glucose concentrations with duration of treatment **A**, association of mean changes in TG concentrations with duration of treatment **B**, association of mean changes in plasma HbAc1 concentrations duration of treatment **C** and mean age **D**, association of mean changes in BMI duration of treatment **E** and mean age **F** and association of WC duration of treatment **G** and mean age **H**

On the other hand, meta-regression analysis revealed that cocoa supplementation decreased glucose (*p* = 0.0042), HBA1c (*p* = 0.0046), TG (*p* = < 0.0001) levels and BMI (*p* = < 0.0001) and WC (*p* = < 0.0001) as the study duration increased. Furthermore, the mean age of study participants influenced HbA1c level (*p* = 0.047), BMI (*p* = 0.0009) and WC (*p* = < 0.0001) (Fig. 7).

### Publication bias

Publication bias assessment was conducted using funnel plots and Egger’s linear regression test. A visual inspection of the funnel plot revealed no publication bias in the majority of included studies (data not shown). Egger’s linear regression test confirmed the absence of publication bias (TC: *p* = 0.4508, LDL: *p* = 0.1974, TG: *p* = 0.9352, glucose: *p* = 0.5855, insulin: *p* = 0.0693, HbA1c: *p* = 0.7794, SBP: *p* = 0.9313, DBP: *p* = 0.5307, BMI: *p* = 0.5923, weight: *p* = 0.1270, WC: *p* = 0.3645). However, for HDL levels (*p* = 0.0214) and HOMA (*p* = 0.0374) Egger's linear regression test confirmed a publication bias. Egger's test has low power when < 10 studies are included; therefore, these results should be interpreted with caution.

### - Quality of the evidence for the outcome using GRADE

GRADE categorized the quality of evidence into four levels: High, Moderate, Low and Very Low quality, ranging from confidence that the true effect approximates the estimate of the effect to that the true effect is likely to be substantially different from the estimate of the effect. All outcomes were judged as moderate or low quality of evidence, based on the GRADE approach, due to methodological limitations (risk of bias), the imprecision of pooled effects related to a small number of participants and publication bias since small studies with negative results were missing (Table 3).

**Table 3:** Summary of main results

Outcome	Studies	Participants	Effect estimate	*P* value	Quality of evidence (GRADE)
TC	10	404	−0.14 [−0.45, 0.16]	0.3606	⊕ ⊕ ⊕ ⊝ Moderate^1^
LDL	10	412	−0.21 [−0.48, 0.05]	0.1170	⊕ ⊕ ⊕ ⊝ Moderate^1^
HDL	10	404	0.07 [−0.01, 0.14]	0.0876	⊕ ⊕ ⊝ ⊝ Low^1,3^
TG	10	404	−0.21[−0.40, −0.02]	0.0333	⊕ ⊕ ⊕ ⊝ Moderate^1^
FPG	8	274	−0.20 [−0.68, 0.28]	0.4106	⊕ ⊕ ⊕ ⊝ Moderate^2^
Insulin	7	290	−0.97 [−3.20, 1.27]	0.3969	⊕ ⊕ ⊕ ⊝ Moderate^2^
HOMA	7	272	−0.21 [− 0.47, 0.04]	0.0962	⊕ ⊕ ⊝ ⊝ Low^2,3^
HbA1c	3	179	−0,48 [− 1,28, 0.33]	0.2434	⊕ ⊕ ⊝ ⊝ Low^1^,^2^
SPB	8	317	− 2.16 [−5.34, 1.02]	0.1839	⊕ ⊕ ⊕ ⊝ Moderate^2^
DPB	8	317	− 1.46 [−3.70, 0.78]	0.2015	⊕ ⊕ ⊕ ⊝ Moderate^2^
Weight	9	311	0.91 [−0.01, 1.83]	0.0532	⊕ ⊕ ⊕ ⊝ Moderate^2^
WC	8	331	−1.58 [−4.14, 1.01]	0.2312	⊕ ⊕ ⊝ ⊝ Low^1,2^
BMI	8	328	−0.28 [− 0.82, 0.27]	0.3195	⊕ ⊕ ⊝ ⊝ Low^1, 2^

^1^Methodological limitations (risk of bias)

^2^Downgraded due to imprecision (95% confidence interval of the pooled effect includes no effect and negative effect), lack of precision < 400 participants

^3^Publication bias: Literature were searched exhaustively. Funnel plots were conducted and showed asymmetry (small studies with negative results were missing)

## Discussion

The results of this meta-analysis of thirteen studies, including sixteen arms, indicated that administration of cocoa significantly had no significant effects on arterial pressure, glycemic status markers and anthropometric parameters. However, cocoa supplementation reduced triacylglycerol levels, without altering the other parameters of the lipid profile. In addition, cocoa supplementation decreases cholesterol levels in patients with dyslipidemia and diabetes. Cocoa supplementation compared to chocolate supplementation revealed a decrease in total cholesterol. On the other hand, subgroup analysis suggested that chocolate supplementation decreased serum glucose levels, DBP and BMI when compared to studies that supplemented with cocoa. The meta-regression analysis suggested cocoa supplementation decreased glucose, HbA1c, TG levels and BMI and WC as the study duration increased.

The significant reduction in triacylglycerol levels promoted by cocoa supplementation was found in other studies corroborating ours. A meta-analysis published in 2021, using studies with healthy people, found that chocolate supplementation reduced triacylglycerol levels [55]. Similar to that, two meta-analyses focusing on cardiovascular health [56, 57] found a significant reduction compared to placebo. However, a meta-analysis conducted in 2024 [16] with studies in adults with and without established comorbidities and a meta-analysis in 2021 [58] in patients with type 2 diabetes found no evidence of the effect of cocoa consumption on triacylglycerol levels. In addition, our meta-regression analysis, we found that studies with longer duration showed a greater reduction in triacylglycerol levels than studies with shorter duration. The flavanols, particularly the procyanidins, found in cocoa, are potent lipase inhibitors in vitro [59, 60]; reduce acute postprandial [60] and fasting plasma triglycerides [61] and increase fecal lipid excretion [62] in animals and humans. It has been demonstrated that cocoa reduces blood triglycerides and lipid accumulation visceral and hepatic in animal models [61–65].

Corroborating our findings, other studies have not found significant changes in HDL and LDL cholesterol levels promoted by supplementation with cocoa products, such a systematic review conducted by Tan et al. 2021[55] in healthy human subjects. On the other hand, the meta-analysis published in 2024 by Arisi et al. [16] found a reduction in LDL in unhealthy individuals, with no such effect in healthy individuals promoted by cocoa supplementation. The same reduction effect was verified for patients with diabetes in a meta-analysis developed by Darand et al. using cocoa/dark chocolate in observational studies in 2021[58]. Furthermore, meta-analyses conducted by Hooper et al. in 2012, with chocolate, cocoa, and flavan-3-ols [56] and Lin et al. using cocoa flavanol in 2016 [57] revealed an increase in HDL cholesterol concentration.

We suggest that the discrepancies found in these studies for cholesterol metabolism may be due to the different health conditions of the individuals participating in the studies and also the type of cocoa product that was supplemented. Corroborating this hypothesis, in our subgroup analysis we found that cocoa supplementation was able to reduce cholesterol levels in patients with dyslipidemia and diabetes. Additionally, cocoa supplementation revealed a decrease in total cholesterol compared to chocolate supplementation.

Our results found that cocoa supplementation in individuals with metabolic syndrome and associated diseases was not able to promote a significant reduction in the glycemic profile. However, the results found in the literature are discrepant. A meta-analysis conducted in healthy adults or those with comorbidities found a reduction in blood glucose levels promoted by supplementation with cocoa products [16]. However, in the meta-analysis conducted by Hooper et al. [66] in adult participants with some risk of cardiovascular disease, did not observe a reduction in blood glucose with supplementation with cocoa products.

The metaregression analysis conducted in this study found that cocoa products were more efficient in improving the glycemic profile in a longer study. The meta-analysis conducted by Chen et al. (2022) [17], in adults with type II diabetes mellitus, also found that in long-term studies, cocoa products reduced serum glucose, and that the same effect was not found in short-term studies. Other studies in cells and healthy individuals have also indicated that the intake of cocoa products promotes long-term glucose homeostasis [67, 68].

Several mechanisms are suggested for improving glucose homeostasis, such as cocoa flavanols, through the slowing of digestion and absorption of carbohydrates in the intestinal tract [69], by inhibiting the digestive enzymes α-amylase and α-glucosidase, protect pancreatic b cells, the inhibition of the glucose transporter, glucose transporter 2 (GLUT2), increase insulin secretion, modulate intracellular signaling pathways and genes involved in gluconeogenesis and glycogenesis, and the promotion of the secretion of glucagon-like peptide 1 (GLP-1) [59, 70, 71].

The results of the current study showed that cocoa supplementation not altered blood pressure. In agreement with these findings, the systematic review conducted by Tan et al. [72], which evaluated the effects of cocoa and its derivatives in healthy individuals also reported no significant effect of chocolate and cocoa on BP. Similarly, the meta-analysis by Darand et al. [73], conducted in patients with type 2 diabetes mellitus, found that cocoa supplementation had no effect on BP. However, contrasting results were observed in the meta-analyis by Amoah et al. [74], which included individuals with normal or elevated BP and in the study by Jafarnejad et al. [75], conducted in middle-aged and elderly individuals with or without metabolic syndrome and related conditions; both studies reported a BP-lowering effect of cocoa. The presence of potential sources of between-study heterogeneity in some of these meta-analyses could explain these different results.

Subgroup analysis revealed a reduction in DBP in the chocolate group, corroborating the meta-analysis developed by Tanghe et al. 2021 [35]. One of the most well-established mechanisms by which chocolate may reduce DBP involves the action of flavonoids, which promote endothelium-dependent vasodilation [76], thereby impacting peripheral vascular resistance. The observed decrease in DBP may be explained by its close association with peripheral vascular resistance, in contrast to systolic blood pressure (SBP), which is more strongly influenced by cardiac output and the capacity of proximal arteries [77].

Our study corroborates the meta-analysis developed by Kord-Varkaneh et al. in 2019 [78] that showed no significant effect on anthropometric measurements after the treatment with cocoa. However, the duration of the studies was an important determinant in verifying the favorable effects on anthropometric measures. Studies suggest that consumption of dietary flavanol-containing substances could reduce BMI and WC [33, 79]. The mechanisms of the anti-obesity potential of flavonols are well investigated, although the exact mechanisms have not yet been clarified. Flavanols have shown a thermogenic effect [80]. They are able to downregulate expression of enzymes involved in biosynthesis of fatty acids, cholesterol and lipogenesis [81, 82]. Additionally, they act on the pancreatic enzymes lipase and amylase [62] and can suppress appetite through increasing GLP-1 and decreasing ghrelin concentration [69, 83].

Plant polyphenol interventions often produce small, domain-specific changes rather than broad metabolic reversal. A meta-analysis of artichoke supplementation found modest improvements in lipids, blood pressure, and insulin resistance but not fasting glucose, very similar to our pattern of selective cardiometabolic effects [18]. Furthermore, a recent meta-analysis on sumac (Rhus coriaria) showed improvements primarily in glycemic and lipid parameters, but not in inflammatory markers, again reflecting the pattern that phytochemical interventions often target limited metabolic domains [20]. These findings may explain the selective effect of cocoa on triglycerides in a broader phytochemical context.

In our study, we observed significant differences in some metabolic parameters when changing the type of cocoa product—chocolate or cocoa. This can be explained by the difference in bioavailability of the flavanols recorded for cocoa and chocolate. Products containing cocoa have a much higher value of procyanidins when compared to chocolate [84]. Furthermore, chocolate contains sugar and fat that may counteract flavanol benefits and reflecting the high between-study heterogeneity.

The strengths of this meta-analysis include the synthesis of evidence from RCTs examining MetS-related parameters, detailed subgroup analyses, and a comprehensive assessment of potential biases. Furthermore, our subgroup analysis suggested that cocoa interventions may have effects on biomarkers of lipid and glucose metabolism, depending on existing comorbidities, intervention duration, and types of cocoa supplementation.

However, there are several potential limitations to our study. The heterogeneity of the studies included in this meta-analysis is the most important limitation. The included trials were conducted in individuals with different health conditions (obesity, type 2 diabetes, hyperlipidemia and hypertension, NAFLD, and metabolic syndrome). Another limitation is the difference in duration and type of cocoa supplementation. Different forms and amounts of cocoa, including flavanol-rich cocoa, procyanidin, cocoa powder, dark chocolate, and cookies, were included. In addition, our meta-analysis was limited by the small number of publications in the subgroup analyses, which resulted in considerable clinical and methodological heterogeneity between studies. Furthermore, there was a time limit for searching the studies included in this meta-analysis, which may have contributed to limited data in the subgroup analyses. These limitations of our review were reflected in the GRADE assessment, which presented all outcomes as having moderate or low quality of evidence; therefore, our findings were interpreted conservatively.

## Conclusion

Our systematic review and meta-analysis showed that the consumption of cocoa products may contribute modestly to triglyceride reduction, but evidence for broader metabolic benefit remains limited and heterogeneous. Further investigations are needed to elucidate the exact mechanisms of action of cocoa, as well as the appropriate dose, type of supplementation, duration, and clinical conditions of the individuals.

## Supplementary Information

Below is the link to the electronic supplementary material.

Supplementary file1 (DOCX 20 KB)

## Data Availability

No datasets were generated or analysed during the current study.
