# Supplementary material for: The effects of cocoa products in individuals with metabolic syndrome and related diseases: a systematic review and meta-analysis
Source: J Diabetes Metab Disord. 2026 Mar 17;25(1):116. doi: 10.1007/s40200-026-01914-7 (PMC12996486; doi:10.1007/s40200-026-01914-7)
Supplement: Supplementary file 1 — Supplementary file1 (DOCX 20 KB) [file 40200_2026_1914_MOESM1_ESM.docx]

SUPPLEMENTARY MATERIAL

# Supplementary Table 1. Search keywords categorized by intervention, outcomes, and study design

| **Intervention** | **Outcomes** | **Study Design** |
| --- | --- | --- |
| cocoa OR cacao OR chocolate OR Cocoa Powder OR Theobroma cacao | “syndrome X” OR “Metabolic syndrome” OR ‘MetS’ OR ‘Met-Syn’ OR  ‘metabolic syndrome X’ OR ‘cardio metabolic risk factor’ OR ” blood  pressure” OR “blood sugar” OR “body mass index” OR “waist circumference” OR ‘insulin resistance syndrome’ OR “cardio metabolic” OR “cardiometabolic” OR Elevated blood pressur OR  OR obesity OR  Cardiometabolic Risk Factor OR 'lipid” OR “cholesterol” OR “triglyceride,” “blood glucose” | “Clinical Trial” OR ”Randomized Clinical Trial” OR ”Randomized Clinical Trials” OR “Randomized Controlled Clinical Trial” OR “Randomized Controlled Clinical Trials” OR “Randomized Controlled Trials” OR “Randomized Controlled Trial” OR “Randomised Controlled Trial” OR “Randomised Controlled Trials” |
